# Coal Tar and Paving Products

**DOI:** 10.1289/ehp.114-a210a

**Published:** 2006-04

**Authors:** Paul A. Schulte

**Affiliations:** Education and Information Division, National Institute for Occupational Safety and Health Centers for Disease Control and Prevention Cincinnati, Ohio, E-mail: pas4@cdc.gov

The National Institute for Occupational Safety and Health (NIOSH) has a partnership relationship with the asphalt paving and roofing industries and their associated unions. Our partners saw in the *EHP* Focus article “Paving Paradise: The Peril of Impervious Surfaces” (Frazer 2005) the statement on page A459: “Asphalt is one concern, as it contains coal tar pitch, a recognized human carcinogen .... ” Our partners asked us if we could help them address this statement.

By definition, asphalt is a petroleum product and contains no coal tar. However, some pavement-repair products and sealants may contain coal tar. NIOSH did not find any evidence of coal tar in U.S. asphalt in our hazard review *Health Effects of Occupational Exposure to Asphalt* (NIOSH 2000.
